# Lower extremity soft tissue reconstruction and amputation rates in patients with open tibial fractures in Sweden during 1998–2010

**DOI:** 10.1186/1471-2482-14-80

**Published:** 2014-10-16

**Authors:** Ulrika Tampe, Rüdiger J Weiss, Birgit Stark, Pehr Sommar, Zewar Al Dabbagh, Karl-Åke Jansson

**Affiliations:** 1 Department of Molecular Medicine and Surgery, Section of Orthopaedics and Sports Medicine, Karolinska Institutet at Karolinska University Hospital, SE-17176 Stockholm, Sweden; 2 Department of Molecular Medicine and Surgery, Section of Plastic Surgery, Karolinska Institutet, Karolinska University Hospital, Stockholm, Sweden

**Keywords:** Skeletal trauma, Tibial shaft fracture, Open fracture, Amputation, Lower extremity reconstruction, Limb salvage, Soft tissue injury

## Abstract

**Background:**

The rates of soft tissue reconstruction and amputation after open tibial fractures have not been studied on a national perspective. We aimed to determine the frequency of soft tissue coverage after open tibial fracture as well as primary and secondary amputation rates.

**Methods:**

Data on all patients (> = 15 years) admitted to hospital with open tibial fractures were extracted from the Swedish National Patient Register (1998–2010). All surgical procedures, re-admissions, and mechanisms of injury were analysed accordingly. The risk of amputation was calculated using logistic regression (adjusted for age, sex, mechanism of injury, reconstructive surgery and fixation method). The mean follow-up time was 6 (SD 3.8) years.

**Results:**

Of 3,777 patients, 342 patients underwent soft tissue reconstructive surgery. In total, there were 125 amputations. Among patients with no reconstructive surgery, 2% (n = 68 patients) underwent amputation. In an adjusted analysis, patients older than 70 years (OR = 2.7, 95%, CI = 1.1-6) and those who underwent reconstructive surgery (OR = 3.1, 95% CI = 1.6-5.8) showed higher risk for amputation. Fixations other than intramedullary nailing (plate, external fixation, closed reduction and combination) as the only method were associated with a significant higher risk for amputation (OR 5.1-14.4). Reconstruction within 72 hours (3 days) showed better results than reconstruction between 4–90 days (p = 0.04).

**Conclusions:**

The rate of amputations after open tibial fractures is low (3.6%). There is a higher risk for amputations with age above 70 (in contrast: male sex and tissue reconstruction are rather indicators for more severe soft tissue injuries). Only a small proportion of open tibial fractures need soft tissue reconstructive surgery. Reconstruction with free or pedicled flap should be performed within 72 hours whenever possible.

## Background

Epidemiological studies have shown an incidence rate of 11.5 per 100,000 person-years for open long bone fractures [1]. A large proportion of these fractures are open tibial fractures [1-4]. Open tibial fractures are associated with a high rate of complications such as compartment syndrome, mal-union, non-union, osteomyelitis, and amputation [5,6]. Gustilo type III open fractures are the most severe, involving extensive soft-tissue trauma, and complex fracture patterns [7]. Some of these injuries can be managed with salvage procedures involving either a pedicled flap or microvascular free flap for coverage of soft tissue defects. An alternative to reconstruction of such severe injuries is amputation.

The choice of limb salvage vs. amputation has been a topic for discussion in many studies [6-10]. Both procedures are associated with complications. Sequelae after limb salvage include osteomyelitis, non-union, or flap loss [5,9].

Bosse et al. reported that patients with leg-threatening injuries had a similar clinical outcome after limb salvage compared with amputation at 2-years follow-up [8]. Others have shown that limb salvage resulted in lower costs and higher utility compared with amputation [11]. Saddawi-Konefka et al. documented a trend towards limb salvage rather than amputation due to an improvement in surgical techniques for soft tissue reconstruction [6].

The recommended standard treatment for Gustilo type III open fractures is stabilization of the fracture and early soft tissue coverage (preferably within 72 hours). This concept was introduced by Godina [12] and has been further developed and evaluated [13-15].

So far, there have been no reports on a national level analysing the rates of limb salvage and amputation in patients with open fractures of the lower limbs. Therefore, we aimed to study the frequency of these surgical procedures in patients with open tibial fractures using Swedish national registries.

## Methods

### Source of data

Data were obtained from the Swedish National Patient Register [16], which covers more than 98% of all hospital admissions in Sweden [17]. A 10-digit national registration number for each individual in Sweden allows epidemiological studies on a nationwide basis. The Register includes e.g. data on diagnosis, surgical procedure codes, and demographic data for each hospital admission in Sweden. Diagnoses are coded according to the International Classification of Diseases (ICD). We extracted data from the Register on all hospital admissions and re-admissions of patients (> = 15 years of age) with the following diagnoses: open fractures of the proximal tibia (S82.11), the tibial shaft (S82.21), and the distal tibia (S82.31). No exclusions were made. The study period was 1998–2010. Fracture incidence rates per 100,000 person-years were calculated by using data from the Total Population Register.

Mechanisms of injury were collected from ICD E-codes and divided into 5 categories: fall on the same level, unspecified fall, fall from height, motor vehicle accident (MVA), and miscellaneous. Fall from height and MVA were considered as high energy mechanisms for the risk factor analysis. Fixation methods were grouped into 6 categories: intramedullary nail, plate, closed reduction/cast, external fixation, combination of external fixation and other definitive fixation, and miscellaneous. Reconstructive plastic surgical procedures were grouped into 3 categories: free flaps (ZZQ), pedicled flaps (ZZS), and skin graft only (ZZA00). Timing of free and pedicled flaps was registrered and analysed in categories: reconstruction within 72 hours (3 days), reconstruction within 4 to 90 days and reconstruction after 90 days. Amputations were analysed as follows: transfemoral amputation (NFQ19), knee disarticulation (NGQ09), and transtibial amputation (NGQ19). Ankle disarticulation and partial foot amputation were regarded as one group (NHQ). Amputation was defined as early if performed within 3 months and late after 3 months. The etiology of amputation was registered as severe acute injury, infection/osteomyelitis, pseudarthrosis, high age and other/unknown. The study was approved by The Regional Ethical Review Board (2011/1280-32).

### Statistics

The Welsh 2 Sample t-test was used to calculate differences for mechanisms of injury, sex, and mean age of the amputated as compared to the non-amputated patients. Amputation rate related to timing of reconstruction was analyzed with Fisher’s exact test for count data. The differences between amputation rates after reconstructive procedures were calculated using Fisher’s exact test for count data and Bon Ferroni correction. Logistic regression analysis was used to assess the risk of amputation within 3 months after the fracture. Odds ratios (ORs) and the associated 95% confidence intervals (CI) are presented. The crude results were adjusted for age, sex, mechanism of injury, and surgical procedure. Logistic regression with a binomial logit link function was applied. The results were considered statistically significant for p-values < =0.05. The statistical software used was R. R is available as free software, http://www.r-project.org.

## Results

### Patients

**Figure 1:**
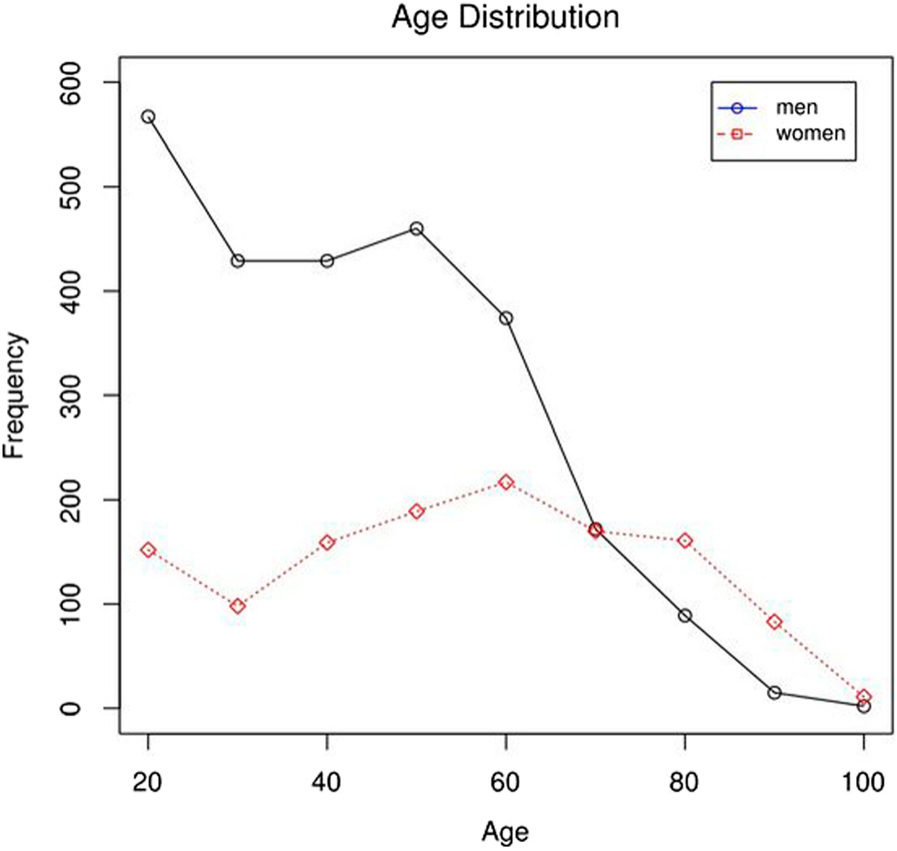
Age distribution of men and women with open tibial fractures in Sweden during 1998-2010.

During the study period, 3,777 patients (67% males) were admitted to a Swedish hospital due to open tibial fractures (Table 1). The majority (3,704 patients, 98%) had a unilateral fracture and 73 patients had bilateral fractures (2%). Most fractures were located in the tibial shaft (60%). The mean age of the patients at admission was 47 (SD 20) years (males 42 [SD 20] and females 55 [SD 22]) (Figure 1). The mean follow-up time was 6 (SD 3.8) years.

**Table 1:** Patients with open tibial fractures in Sweden during 1998-2010

	**All patients**		**Males**		**Females**	
	**n**	**%**	**n**	**%**	**n**	**%**
	3,777	100	2,537	67	1,240	33
**Localization**						
Proximal	540	14	329	61	211	39
Shaft	2,277	60	1,593	70	684	30
Distal	960	26	615	64	345	36
**Mechanism of injury**						
Fall on same level	791	21	359	45	432	65
Fall from height	414	11	304	73	109	27
Fall unspecified	229	6	138	60	91	40
Motor vehicle accident	1,631	43	1,191	73	440	27
Miscellaneous	712	19	544	76	168	24
**Fixation method**						
Intramedullary nail	1,212	32	850	70	362	30
Plate	325	9	188	58	137	42
Closed reduction, cast	145	4	78	54	67	46
External fixation	294	8	212	72	82	28
Combination external fixation and other methods	815	22	592	73	223	27
Miscellaneous	986	26	615	62	371	38

**Figure 2:**
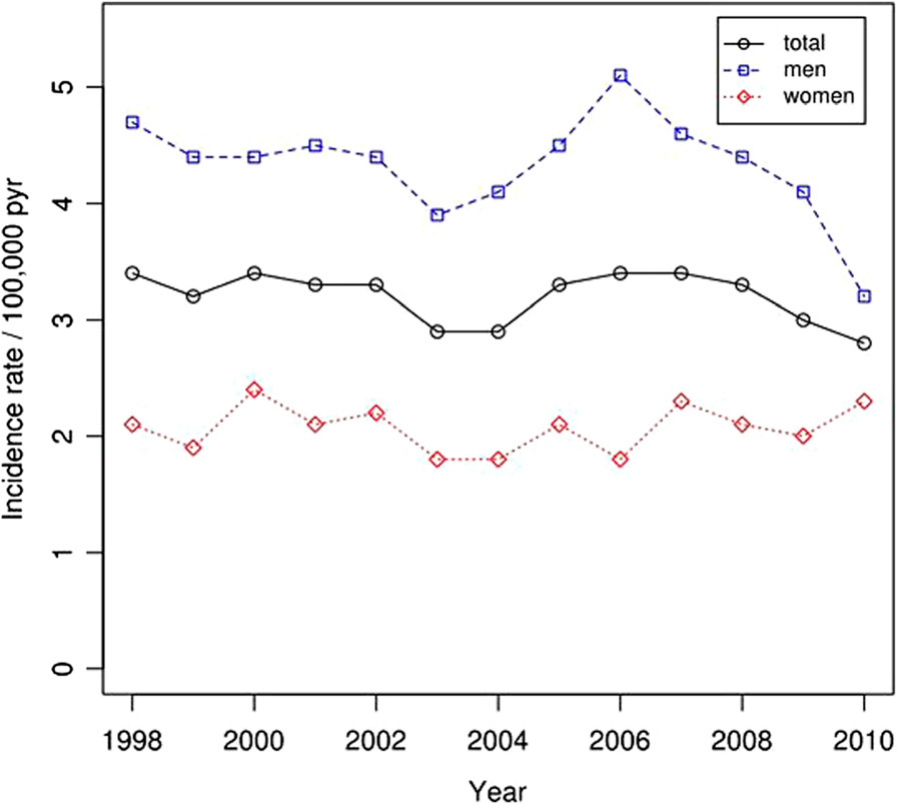
Incidence rate of open tibial fractures per 100,000 person-years.

The most common cause of injury was a MVA (43%), followed by a fall on the same level (21%). Fractures after MVA and falls from height were mainly seen in males (both 73%). Fractures after fall on the same level had a more even distribution (females, 55%) (Table 1). The mean age was higher for females in all age-groups (p < 0.01) (data not included).During the study period, the incidence rate of open fractures was ranging between 2.8-3.4 per 100,000 person-years, and did not show any statistically significant change over time. The incidence rate was higher for males compared with females (p < 0.001) (Figure 2).

### Surgical procedures

The most common fixation method was an intramedullary nail (32%) as the only method. Combinations of external fixation and other methods were used in 22% of the patients. Plate as the only method was used in only 9% of the cases (Table 1).

A total of 342 patients (9%) underwent soft-tissue reconstructions. There were 102 free flaps and 83 pedicled flaps as some patients had more than one procedure. There were 166 patients treated with skin grafts only (Table 2). Reconstructive surgery with free or local flap was performed within 3 days after the injury in 27 patients of whom no one was amputated within the study period. Between 4 and 90 days, there were 97 reconstructions of which 12 (12%) went to amputation. There was a significant difference in amputation rate between the two groups (p = 0.04). Approximately 50% of reconstructions with free or local flap were performed within 10 days, and between day 4 and 7 there were 24 reconstructions, of which 3 went to amputation.

**Table 2:** Surgical procedures after open tibial fractures among 3.777 patients, n = cases

	**All patients**	
	**n**	**%**
**Flaps**		
Total	342	100
Free flaps	102	30
Pedicled flaps	83	22
Skin graft only	166	48
**Amputations**		
Total	125	100
Transfemoral	30	24
Knee disarticulation	17	14
Transtibial	74	59
Ankle and foot	4	3

During the study period, 93 out of 3,777 patients underwent an amputation. In total, there were 125 amputations. The majority (59%) were transtibial amputations. At first admission, an amputation was performed in 43 patients. Early amputations were done in 63 patients and late amputations in 30 patients. Data showed that amputation was performed on day one in 28 patients. The etiology for an amputation within first admission was in 34 cases the acute injury; associated injuries like vascular damage (n = 11), severe open injuries, open foot injuries, nerve injuries, crush injuries, multiple fractures and dislocations. Other causes were acute infection (n = 2), patients with a high age (n = 13) and other/unknown reason (n = 2). In cases where amputation was performed later then first admission, the etiology was infection/osteomyelitis (n = 20), initial history of polytrauma with severe associated injuries (n = 8), pseudarthrosis (n = 6), and other/unknown reason (n = 8).

Amputated patients were older than non-amputated patients (p < 0.001). The mean age for amputated men was 51 (SD 20) years and for women 70 (SD 19) years. For non-amputated patients the mean age was 42 (SD 18) years for men and 55 (SD 21) years for women.

Patients who did not have soft tissue reconstruction had a lower amputation rate (2%) compared with patients who had reconstructive surgery (7%) (p < 0.001). No significant difference was seen between the three methods of tissue coverage regarding subsequent amputation (p = 0.44) (Table 3).

**Table 3:** Total number of amputations related to previous reconstructive surgery, n = patients

	**No amputation**	**Total amputations**		**Early amputation <3 months**		**Late amputation >3 months**	
	**n**	**n**	**%**	**n**	**%**	**n**	**%**
**Flaps**							
No flap	3,367	68	2	50	2	18	0.5
Free flap	93	9	9	3	3	6	6
Pedicled flap	76	7	10	3	4	4	5
Skin graft only	155	11	7	8	5	3	2
All reconstructions	315	25	7	13	4	12	4

Some patients have undergone several procedures.

### Risk factors for amputation

Logistic regression analysis with the outcome risk for amputation within 3 months after fracture showed higher risk for patients with male sex, age above 70 years and in those who underwent reconstructive surgery. The mechanism of injury did not show any significant association. Regarding fixation methods, methods other than intramedullary nailing as the only method were associated with a significant higher risk for amputation (Table 4).

**Table 4:** Risk factors for amputation within 3 months in patients with open tibial fracture

		**Crude**			**Adjusted**		
		**OR**	**95% CI**	**p**	**OR**	**95% CI**	**p**
**Sex**	Men	1.0	ref		1.0	ref	
	Women	0.9	0.5-1.6	0.78	0.5	0.3-1.0	0.050
**Age-group**	15-60	1.0	ref		1.0	ref	
	61-70	1.4	0.6-2.9	0.45	1.6	0.6-3.5	0.27
	71-80	2.0	0.8-4.4	0.09	2.3	0.9-5.2	0.05
	≥81	5.8	2.9-10.7	<0.001	7.2	3.3-15.2	<0.001
**Flap**	No	1.0	ref		1.0	ref	
	Yes	2.8	1.4-5.0	0.001	3.0	1.5-5.6	0.001
**Mechanism of injury Energy**	Low	1.0	ref		1.0	ref	
	High	1.2	0.7-1.9	0.58	1.3	0.8-2.3	0.32
**Fixation method**	Nail	1.0	ref		1.0	ref	
	Plate	5.1	1.1-25.8	0.034	4.4	1.0-22.6	0.055
	Closed reduction	14.4	3.5-70.6	<0.001	12.1	2.9-60.3	<0.001
	External fixation	10.0	2.8-46.5	<0.001	6.6	1.8-31.3	<0.001
	Combination	6.2	2.0-27.4	0.0047	4.5	1.4-20.0	0.022
	Miscellaneous	12.9	4.6-53.8	<0.001	10.0	3.5-42.0	<0.001

CI = confidence interval, ref = reference value, adjusted for age, sex, reconstructive surgery, mechanism of injury and fixation method.

## Discussion

We found that nearly 10% of all patients with open tibial fractures were reconstructed with a soft tissue flap in Sweden during 1998–2010. The overall risk of amputation in patients with open tibial fractures was low, ranging between 2-10%. Significant risk factors for amputation within 3 months after fracture were age above 70 years and soft tissue reconstruction, as an indicator of a severe injury.

The incidence rate of open tibial fractures per 100,000 person-years was stable during the study period, ranging between 2.8-3.4. This is in line with data from Court-Brown et al. who described an incidence rate of 3.4 per 100,000 person-years for open tibial shaft fractures in Edinburgh [18].

Open tibial fractures are often caused by high energy trauma. Motor vehicle accident was the most common mechanism of injury in our cohort and 2/3 of the fractures occured in males. This is in line with previous observations, as well as a sex difference where females mostly sustained low energy fractures due to simple falls and males were predominant in the group with higher energy trauma [1,18]. The age distribution for males was unimodal with the highest incidence at a younger age (around 20 years). For females this curve had a rather flat form but there was a bimodal tendency, with one peak around 20, and second peak around 60 years of age. We believe, as was shown in other publications, that the peak in younger age-groups represented high energy fractures and in higher ages low energy (osteoporotic) fractures [1,18]. The most common fixation method in this study was an intramedullary nail as only method. This is regarded as gold standard in shaft fractures. A combination of external fixation and other methods was the used in 22% of the cases. External fixation as an initial method that is later converted to nail or plate is commonly used in polytrauma or severe open injuries. The Gustilo-Anderson classification is often used for grading of open fractures [7]. Unfortunately this classification is not available in our national registers. The classification is graded in I-III, depending on the size of the skin laceration, the degree of contamination, the soft-tissue injury, and the fracture configuration. The Gustilo type IIIB injury has extensive soft-tissue damage with periosteal stripping and bone exposure with inadequate soft tissue for bone coverage, and thus often needs some kind of soft tissue reconstruction. In our cohort of nearly 4,000 patients with open tibial fracture, almost 9% obtained some type of flap or skin graft. Court-Brown et al. reported a prevalence of Type III fractures of 45% and almost 45% of these cases were graded as Type IIIB, which means totally 20% Type IIIB [18]. These numbers seem rather high compared to our population where only 9% of fractures were interpreted as Gustilo IIIB. This may reflect differences in the study cohorts, where our study included patients from all Swedish hospitals (around 50 emergency hospitals), and the material shown above from Edinburgh might be more selected.

We calculated the risk of amputation within 3 months and found age >70 years and the occurrence of flap surgery as independent risk factors. Higher age reasonably increases the risk for other diseases that lead to a poorer outcome for limb salvage. In this set-up, we have no data on co-morbidities. The Swedish Patient Register is valid for primary diagnoses to a great extent, especially for trauma and surgical procedures. When it comes to comorbidities data are more unsecure [17]. We have considered this register not to be valid enough to perform statistical analyses on comorbidities.

We thought that mechanism of injury (an indicator of energy level) would be a risk factor for amputation. However, this was not the case in our cohort. This may indicate that low energy fractures diluted the effect by being present in the group of transport accidents. The subdivision of injury mechanisms in only 5 groups might not be sophisticated enough to show an association between energy level and amputation. Among fixation methods, all methods other than nailing as only method were associated with higher risk for amputation. A reason for this may be that nailing as early definitive treatment is mainly used in low energy fractures, Gustilo type I-II.

Reconstructive plastic surgery as a risk factor may indicate a more severe fracture Gustilo type III, ending in a higher amputation rate. In all cases with free flap reconstructions, a substantial amount of lower limb soft tissue is damaged, exposing the fracture site. Extensive soft tissue deficiency should be considered as a negative prognostic sign for healing.

We could also differentiate the amputations as early (within 3 months) or late. Early amputations were seen in 63 patients and late amputations in 30 patients. Late amputations are considered to have a poorer outcome with more complications [9].

We found the amputation rate after free flap surgery similar to local flap surgery (9% and 10% respectively). For patients without flaps, the amputation rate was 2%. In a review by Saddawi-Konefka et al. the authors reported failure rates for pedicled flaps of 8% and 5% for free flaps [6]. In a study from the LEAP group, the authors found that pedicled flaps were associated with 4.3 times more wound complications requiring operative intervention than free flaps, in one sub-group of severe injuries [19]. Pedicle flaps might be constructed by tissue in the zone of injury affecting complication and amputation rate. This predisposing risk factor highlightened the important pre-operative decision making according to the reconstructive plastic ladder. Timing of flap surgery has earlier been shown to be of great importance for successful reconstruction [12]. Godina showed in his study that reconstruction within 72 hours (3 days) showed better results than reconstruction within 4–90 days or later. In this study results are similar. None of the patients that had reconstructive surgery within 3 days was amputated. The goal for most orthopedic and plastic surgeons is to perform reconstruction within 3 days and the time should not exceed one week [20]. We conclude that this goal is important to achieve whenever possible.

The amputation rate after attempted limb salvage varies between 4-40% in the literature [6,9,14,19,21]. There is an ongoing discussion about the decision between limb salvage and amputation in patients with severe lower limb trauma. We know that the complication rate is higher with attempted limb salvage, but self-reported results are similar after 2 years [9]. To evaluate complications is important, not only for prevention, but also for the possibility to give patients accurate information about lower limb reconstruction.

The major shortcomings of this study are the following: this is a register study with all it’ s advantages and disadvantages. No further investigation of medical records was performed. We have no data on the Gustilo classification, as this variable is not included in the Register. Comorbidities and injury mechanisms may not be valid enough to perform statistical analysis, as mentioned above. A further limitation is the relatively small amount of severe injuries that require reconstructive surgery. Therefore, more complex associations are difficult to detect statistically. However, the amputation rate and frequency of soft tissue coverage after open tibial fracture has not been studied previously on a national basis. Thanks to data obtained from Swedish registers, we have been able to study the epidemiology and outcome of open tibial fractures over a period of 13 years. This patient material covers nearly all hospital admissions in Sweden and we therefore consider the results representative for a population of 9 million inhabitants [17].

The number of patients in Sweden with a severe injury of the lower limb is low. In this material, only 13 limb reconstructions for open tibial fracture were performed every year. Knowledge about outcome of different treatment strategies is crucial to manage these patients properly and provide them with appropriate information on prognostic aspects. To be able to ensure best treatment, these patients with severe open tibial fractures should be treated at dedicated trauma units by multidisciplinary teams with both orthopedic and plastic surgeons. This information may therefore be important to health providers to plan the appropriate and cost-effective management of patients with these severe injuries.

In Sweden, a new fracture register has been developed recently in order to improve the quality of health care (http://www.registercentrum.se). This is promising because it is now possible to follow-up these uncommon and critical patients with severe injury of the lower limb.

## Conclusion

The rate of amputations after open tibial fractures is low (3.6%). There is a higher risk for amputations with age above 70 (in contrast: male sex and tissue reconstruction are rather indicators for more severe soft tissue injuries). Only a small proportion of open tibial fractures need soft tissue reconstructive surgery. Reconstruction with free or pedicled flap should be performed within 72 hours whenever possible.

## Competing interests

The authors declare that they have no competing interests.

## Authors’ contributions

UT and KÅJ: planning, data analysis, statistics, writing, and editing of the manuscript. RJW, BS, PS, and ZAD: planning and editing of the manuscript. All authors read and approved the final manuscript.

## Pre-publication history

The pre-publication history for this paper can be accessed here:

http://www.biomedcentral.com/1471-2482/14/80/prepub
